# Symbiont community stability through severe coral bleaching in a thermally extreme lagoon

**DOI:** 10.1038/s41598-017-01569-8

**Published:** 2017-05-25

**Authors:** E. G. Smith, G. O. Vaughan, R. N. Ketchum, D. McParland, J. A. Burt

**Affiliations:** Centre of Genomics and Systems Biology, New York University, Abu Dhabi, PO Box 129188 UAE

## Abstract

Coral reefs are threatened by climate change as coral-algal symbioses are currently living close to their upper thermal limits. The resilience of the algal partner plays a key role in determining the thermal tolerance of the coral holobiont and therefore, understanding the acclimatory limits of present day coral-algal symbioses is fundamental to forecasting corals’ responses to climate change. This study characterised the symbiont community in a highly variable and thermally extreme (Max = 37.5 °C, Min = 16.8 °C) lagoon located in the southern Persian/Arabian Gulf using next generation sequencing of ITS2 amplicons. Despite experiencing extreme temperatures, severe bleaching and many factors that would be expected to promote the presence of, or transition to clade D dominance, the symbiont communities of the lagoon remain dominated by the C3 variant, *Symbiodinium thermophilum*. The stability of this symbiosis across multiple genera with different means of symbiont transmission highlights the importance of *Symbiodinium thermophilum* for corals living at the acclimatory limits of modern day corals. Corals in this extreme environment did not undergo adaptive bleaching, suggesting they are living at the edge of their acclimatory potential and that this valuable source of thermally tolerant genotypes may be lost in the near future under climate change.

## Introduction

Marine ecosystems worldwide are threatened by climate change^1^. Coral reefs are particularly vulnerable due to the thermal sensitivity of the symbiotic relationship between the coral host and its algal partner of the genus *Symbiodinium* ^2^. Sustained temperatures of just 1–2 °C above the average annual maxima can cause coral bleaching, a breakdown in the coral alga symbiosis, and can result in coral mortality if bleaching persists^2, 3^. Understanding coral responses to thermal stress and the impact of bleaching on the physiology and ecology of coral-algal symbiosis is fundamental to forecasting the future of reefs.

The genetic identity of the coral’s symbionts influences its thermal tolerance, with a range of thermal physiologies associated with distinct symbiont types^4–7^. For example, within clade D, there are certain species such as *Symbiodinium trenchi* (D1-4, formerly D1a) that are considered thermally tolerant. Hosting a stress-tolerant symbiont type, such as the hardy opportunists belonging to *Symbiodinium* clade D^8^ can increase the coral holobiont’s thermal tolerance by 1–2 °C compared with more thermally sensitive variants including representatives from clade C^4^. However, the enhanced resilience of clade D symbioses can come at an energetic cost, impacting the growth rate of the host^9, 10^. Corals can overcome the challenges associated with hosting a thermally tolerant symbiont by changing their symbiont complement in response to environmental conditions^11–13^. Changes in a coral’s symbiont community can occur through two distinct processes: shuffling or switching. Shuffling describes the alteration of the existing symbiont complement within a coral’s tissue to increase the abundance of the physiologically most suitable symbiont^14, 15^. In contrast, a coral may acquire a more suitable symbiont from the environment, termed switching, which has been observed in transplants^4^ and acute, experimentally-applied thermal stress^16^. Early studies on changes in the symbiont community reported the emergence of clade D symbionts after severe bleaching^17^. While alterations of the symbiont community to clade D has been observed in corals on reefs in the Caribbean^12, 13^, other reef symbiont communities have been shown to remain stable despite bleaching^5, 18, 19^, suggesting local biogeographic and host specific factors may be important^18^.

In order to understand how coral symbioses will respond to climate change, there has been an increasing focus on the coral symbiont communities in present day extreme reefs, particularly in highly variable tidal environments^20–22^. These lagoons and tide pools can experience thermal maxima that are >2.5 °C greater than neighbouring environments^23^. Consequently, corals in variable environments often host symbiont communities that are generally dominated by clade D symbionts while those on more benign neighbouring reefs are dominated by clade C symbionts^20, 21^. The association with clade D symbionts is thought to be fundamental to the greater thermal tolerance of lagoonal/tide pool corals compared to those from stable environments, although there are exceptions^24, 25^. The predominance of clade D in these extreme environments is consistent with observations of clade D symbionts on reefs exposed to anthropogenically elevated temperatures^26^, in corals transplanted to warmer environments^4^ and in corals previously exposed to thermal stress and bleaching^12, 13, 17^.

A notable exception to the widespread occurrence of clade D types in thermally extreme reefs exists in the Persian/Arabian Gulf (PAG). Although present in some regions of the PAG^17, 27, 28^, clade D symbionts are largely absent in corals on the southern coastline of the Gulf^19, 29, 30^. This absence is significant because these corals experience the most extreme reef temperature regime globally, with summer mean monthly maxima exceeding 34 °C annually, and with the highest reported bleaching thresholds in the world^31^. Instead, corals in the southern PAG are dominated by a variant of clade C3, *Symbiodinium thermophilum*, a member of an ancient symbiont lineage that is cryptically distributed outside of the PAG^32^. These symbionts have been suggested to be among the most thermally robust symbionts associated with coral and form symbioses that are stable over time with no shifts to clade D^19^. To date, this symbiont has only been identified in open-water or offshore island reef environments that are characterized by relatively limited diurnal variability in temperature, in marked contrast to the clade D dominated highly variable lagoonal and tidal systems in the northern PAG and elsewhere^20, 21, 27^. The composition of the symbiont community in corals in lagoonal environments of the southern Gulf is unknown, but has important implications given the extreme nature of these environments. Corals in lagoonal systems in the southern PAG are exposed to considerable chronic and acute thermal stress, with long-term summer temperatures on reefs 1.5 °C greater than the adjacent open water locations, and with diurnal temperature ranges exceeding 10 °C^33–35^. It is unclear whether the highly variable thermal regime impacts the stability of the symbiont community, particularly during bleaching events which have altered communities elsewhere^12, 13, 17^.

Corals in southern PAG lagoonal reefs are exposed to conditions at the acclimatory limits of modern day coral symbioses and therefore it is essential to characterise the symbionts responsible for one of the most resilient coral communities in the world. To this end, using an amplicon sequencing approach, the symbiont community composition of a southern PAG lagoonal system was investigated before and after a severe bleaching event to assess whether these reefs are clade D or *S. thermophilum* dominated, and to quantify the community changes in response to one of the highest ever recorded reef temperatures.

## Methods

### Sampling locations

Two lagoon sites were surveyed in this study, each located on the southern coastline of the PAG, in the Emirate of Umm al Quwain (UAQ). The two sites, UAQ1 (25°32′35.6′′N 55°35′29.9″E) and UAQ2 (25°34′06.6″N 55°34′11.3″E) are located 5 km and 2.5 km from the entrance of the lagoon respectively and are dominated by poritids and merulinids. Full descriptions of the coral communities are reported elsewhere^36^.

### Benthic sampling

The coral community was surveyed using an established photoquadrat methodology^37, 38^. Briefly, 0.25 m^2^ quadrats were photographed every 3 m along six 30 m transect tapes, providing a total of 66 images. The images were analysed by CPCe 4.1^39^ with 50 points per image. Each point was classified to different benthic cover types, with corals identified to genus level and bleaching added as a note where necessary. The degree of bleaching was calculated as the proportion of bleached coral points relative to the total number of points for each coral genus. The surveys was performed in June, September and December 2014.

The thermal environment at the two sites was recorded every 5 minutes between June 2014 and February 2015 using HOBO TidBit v2 loggers. Temperature data was also collected (sampling every 30 minutes) at a well-studied open water southern Arabian Gulf reef, Saadiyat, for comparison. Loggers were attached to colonies or directly adjacent (within 30 cm) to ensure representative recording of the temperatures experienced by the coral communities.

### Symbiont communities

Small (0.25 cm^3^) coral fragments were collected from both sites during June (50 samples; pre-bleaching) and December 2014 (59 samples; post-bleaching). Samples were collected from 10 coral genera representing 6 different families. Each sample was placed in a ziplock bag and transferred to liquid nitrogen upon return to the boat. Fragments were stored at −80 °C until extraction.

*DNA extraction*: Fragments were lysed in a Tissuelyser using 7mm beads (22 Hz for 12–30 s). The resulting powder was mixed with an SDS based lysis buffer^40^ (containing RNase) and incubated at 65 °C for 10 minutes. A precipitation buffer (3 M potassium/5 M acetate) was subsequently added and the samples left on ice for 10 minutes. After centrifugation, the supernatant was mixed with 0.8x volumes of carboxylated modified magnetic beads in the presence of a PEG/NaCl buffer^41^ and incubated for 5 minutes. The beads and the supernatant were subsequently separated using a magnetic rack and the magnetic particles washed three times with 80% ethanol before elution with molecular biology grade water. Extracted DNA was stored at −20 °C prior to use.

Amplification of the ITS region was performed using triplicate PCRs with customised Sym_Var primers^30^, modified to include Illumina sequencing primer sequences and a 5 bp inline barcode. A total of 47 unique barcodes (each differing by at least 2 bp^42^) were used. PCR was performed using the high fidelity PrimeStar GXL enzyme according to manufacturer’s recommendations and the following cycling conditions (1 cycle: 98 °C 10 s, 30 cycles: 98 °C 10 s, 57 °C 20 s, 68 °C 60 s, 1 cycle: 68 °C 5 min). DNA concentration in the PCR reactions was quantified using Qubit HS assay and normalised in pools of 47 individuals before purification with Ampure XP beads. Purified pools were divided into three and subjected to a 6 cycle PCR to attach flow cell annealing adapters and 6 bp Illumina Indexes. Each pool (with unique index) was quantified using the KAPA Illumina library quantification kit and normalised to a 2 nM pool. Sequencing was performed by the New York University Abu Dhabi Core Sequencing Facility using a MiSeq 600 cycle v3 kit as part of a larger pool and only the single end read ITS2 region was used for this study^43^. Adapters were removed and sequences trimmed (QC < 25) in Trimgalore prior to passing through the mothur pipeline described by ref. 44, modified for reads obtained through the Illumina MiSeq platform. An important modification to the protocol involved resampling all individuals to the read depth of the lowest sampled individual (n = 1889). This was performed prior to separation of sequences by clade and involved the removal of 9 samples from the dataset due to low read depths (n < 1889). The resampled dataset is available at Dryad (doi:10.5061/dryad.p8f6n). The mothur pipeline parameters used were [trim. seqs; pdiffs = 2, bdiffs = 0, maxambig = 0, minlength = 220][split. abund; cutoff = 3] [screen. seqs; criteria = 85] [get. oturep; label = 0.03]. In addition to the UCHIME chimera removal, the consensus of each of the final OTUs (Operational Taxonomic Units) were blasted and manually verified for the presence of chimeras. Chimeras that were not detected by UCHIME were removed (0.02% of total sequences), the sequences realigned and OTUs reassigned. As OTUs may be characterised by putative pseudogenes^45^, we assessed the secondary structure of each OTU representative using the ‘predict’ function of the ITS2 database^46^. Sequences that has less than 75% conservation in any helix were considered putative pseudogenes.

### Data analysis

Proportional data were arcsin-squareroot transformed prior to analyses. The reported means and confidence intervals were calculated on the arcsin-squareroot transformed data and were subsequently back-transformed^47^. Confidence intervals for proportional data were obtained through percentile bootstrapping of the arcsin-squareroot transformed data in R using 10000 replicates^48^. A three-way Analysis of Similarity (ANOSIM) was used to test for multivariate differences in OTU community structure across the main effects of season, species, and site using Primer-e(v7) software. ANOSIM is a non-parametric multivariate equivalent of an analyses of variance (ANOVA) and is an appropriate test for differences in multivariate community structure such at the zooxanthellae OTU assemblage being examined here^49^. Proportional representation of OTUs present in each sample were arcsin-squareroot transformed prior to analyses to normalize the data, with ANOSIM performed on a Bray-Curtis distance matrix representing the multivariate community. Differences in the abundance of the *S. thermophilum* indel were assessed using the Mann-Whitney U test, and temperature variables (daily mean, daily maximum, daily minimum, daily range) compared between sites using a Welch t test, with the tests performed in Statistica 12. The frequencies of bleached and healthy corals that were abundant at both sites (*Porites* and *Platygyra*) were compared between sites using the Fisher-Freeman-Halton exact test performed in StatXact 11.

## Results

### Thermal environment of the UAQ lagoon

**Figure 1:**
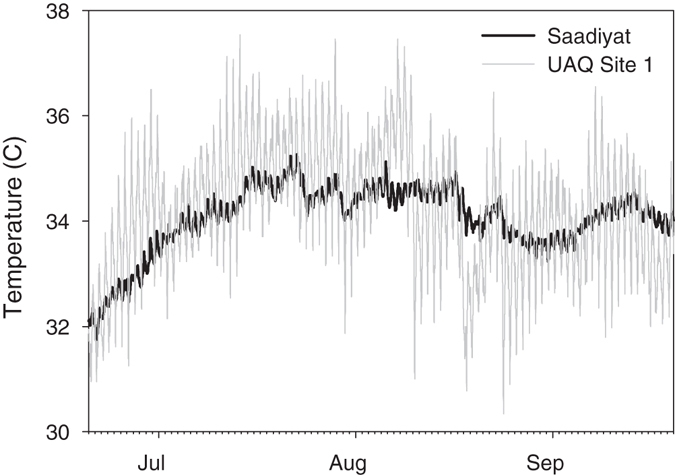
Water temperatures in the Umm Al Quwain lagoon and Saadiyat reef during summer 2014.

The corals in the UAQ lagoon were exposed to mean temperatures >34 °C at each site throughout July and August 2014 (Fig. 1) with a mean daily range of 3 °C. Although the daily mean temperatures for lagoonal UAQ1 and UAQ2 were comparable to that of the off-shore reef (Saadiyat), both the daily range and maximum temperatures were significantly greater at the lagoonal sites compared with the offshore reef (Supplementary Table 1). In the most extreme month, July, lagoonal corals experienced temperatures >34 °C daily (81% of 744 hours), with 36 °C exceeded on a total of 21 days at UAQ1 (mean duration 3.4 hours) and 10 days at UAQ2 (mean duration 1.6 hours). The maximum temperatures experienced were 37.5 °C (UAQ1; 28/07/14 18:35) and 36.9 °C (UAQ2; 07/08/14 16:30) compared to 35.2 °C at Saadiyat reef.

### Widespread bleaching during summer 2014

**Figure 2:**
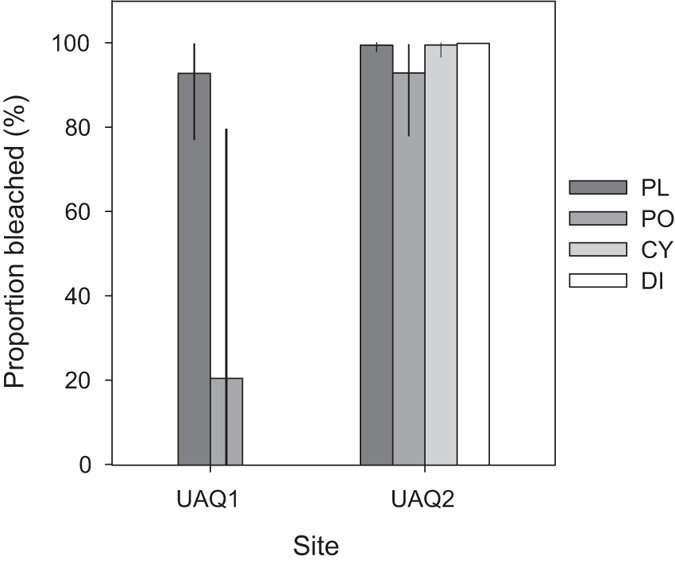
Incidence of bleaching among dominant taxa at two Umm Al Quwain lagoon reefs in September 2014. Only taxa that were present on more than 3 transects and with more than 10 CPCe counts are shown (Supplementary Table 2). Error bars indicate the 95% confidence intervals. PL = *Platygyra*, PO = *Porites*, CY = *Cyphastrea*, DI = *Dipsastraea*.

During summer 2014, widespread bleaching occurred in the UAQ lagoon (Fig. 2). Bleaching was characterised by complete or near complete bleaching of the colonies (equivalent to bleaching scale 3, severe)^50^. Bleaching was documented in all 8 genera recorded on photoquadrat transects, with a total of 92% of CPCe coral points classified as bleached in September 2014. Comparisons of genera abundant at both reef sites showed differences in the degree of bleaching for *Porites* (Fisher-Freeman-Halton Exact test, p < 0.01) and *Platygyra* (Fisher-Freeman-Halton Exact test, p < 0.01) (Fig. 2) between sites.

### Symbiont ITS2 community composition

Assignment at the 97% similarity level grouped sequences into six OTUs. Three of these OTUs accounted for 99.8% of the data and matched ITS2 types C3, A1 and D1 (Accessions: AF499789, AB778581, EU449061) based on the consensus sequences, herein referred to as OTU_C3, OTU_A1 and OTU_D1. The remaining three OTUs were characterised as putative pseudogenes with large deletions (>42 bp) impacting the ITS2 secondary structure. As there is no framework to incorporate OTUs comprised exclusively of putative pseudogenes into *Symbiodinium* community analyses, we excluded these OTUs (0.2% of total reads; Supplementary Data 1 & 2) from the analyses. A total of 75639 and 87362 reads were used in the final analyses from June (pre-bleaching) and December (post-bleaching), respectively; 44% of the reads were from site UAQ1 and 56% from UAQ2.

**Figure 3:**
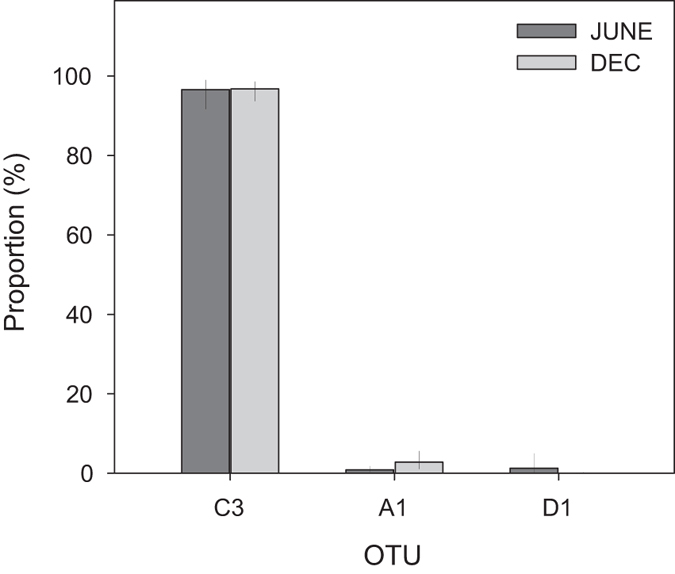
Mean proportion of different ITS2 OTUs in June and December 2014. Error bars show the 95% confidence intervals.

**Figure 4:**
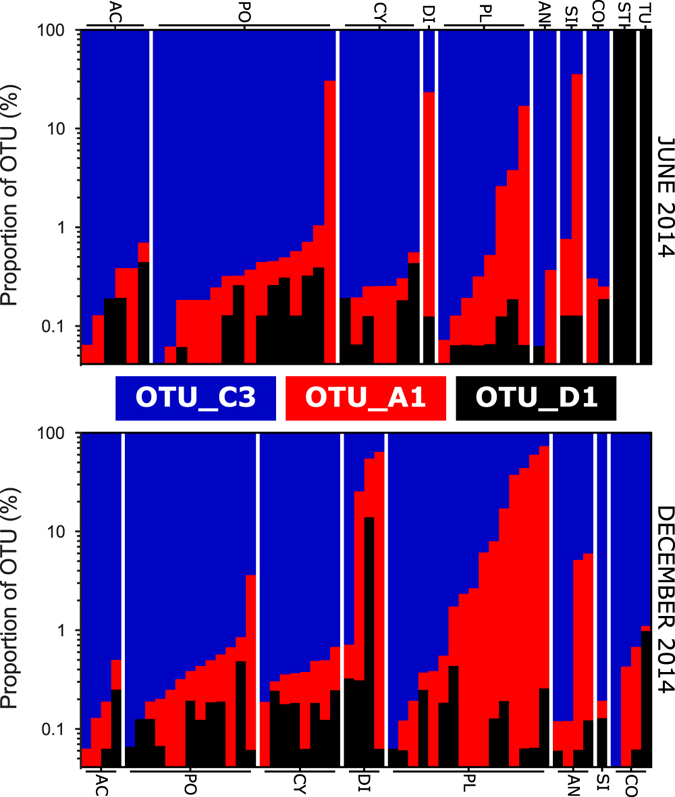
Distribution of ITS2 OTUs within and between Umm Al Quwain lagoon corals sampled in June (upper panel) and December (lower panel) 2014. Each bar represents an individual colony, and these are grouped by genus. Note: vertical axes are log scaled. Labels above and below plots indicate the relevant genera (AC = *Acropora*, PO = *Porites*, CY = *Cyphastrea*, DI = *Dipsastrea*, PL = *Platygyra*, AN = *Anomastrea*, SI = *Siderastrea*, CO = *Coscinaraea*, ST = *Stylophora*, TU = *Turbinaria*).

There was no significant difference in the composition of the symbiont community before and after the mass bleaching event (Fig. 3). A three-way ANOSIM showed that there were no differences in multivariate OTU community structure between the main effects of seasons (R = 0.03, p = 0.72), sites (R = 0.05, p = 0.22), nor species (R = 0.08, p = 0.14). At both time points, the reefs of UAQ were dominated by OTU_C3 (June: 97%; December: 97%) with OTU_D1 compromising <1% in both months, and OTU_A1 present at 1% and 3% in June and December, respectively. OTU_C3 was found in all samples, while OTU_A1 was observed in 91% and 93%, and OTU_D1 in 68% and 72% of samples during June and December, respectively (Fig. 4). In only two genera, *Stylophora* and *Turbinaria* (3% of colonies sampled overall), was OTU_D1 more abundant than OTU_C3. To facilitate comparisons with previous studies, the proportion of ITS2 types within clade D dominated individuals was assessed to identify subdominant ITS2 sequences. As the nomenclature was based on DGGE, only ITS2 types comprising of >10% were considered accounting for the limits of detection of DGGE technology^51, 52^. As such, the *Stylophora* individuals hosted a novel D1-18 (where ‘18’ represents a new ITS2 sequence type, involving 2 substitutions of the D1 sequence; Supplementary Data 3) and *Turbinaria* hosted D1-4-6.

**Figure 5:**
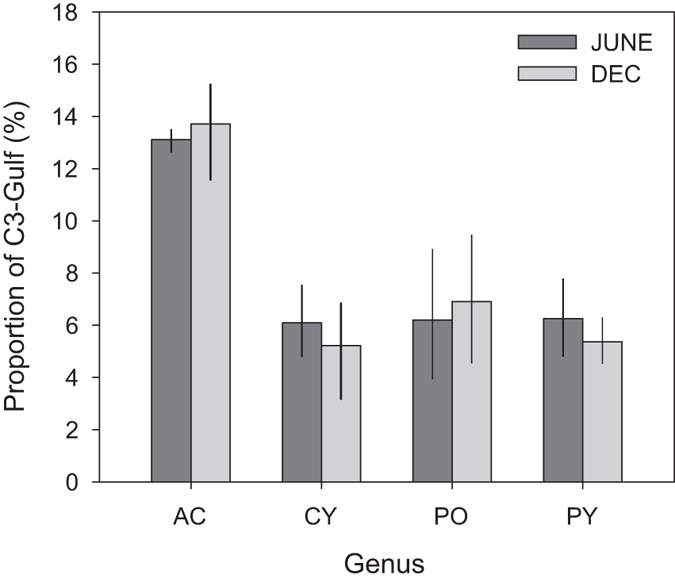
Proportion of C3 reads containing the 8 bp C3-Gulf insertion characteristic of *Symbiodinium thermophilum* collected in June and December 2014. Error bars show the 95% confidence intervals. AC = *Acropora*, CY = *Cyphastrea*, PO = *Porites*, PL = *Platygyra*.

The ITS region is a multicopy marker and consequently, multiple intragenomic ITS2 variants may be present in a single individual. Previous studies on ITS2-type C3 in the PAG have used a characteristic ITS2 intragenomic variant (termed C3-Gulf) to identify the presence of *S. thermophilum* in reefs located in the PAG, Gulf of Oman and Red Sea^19, 32^. The C3-Gulf sequence, although present at lower abundance compared to the dominant C3 sequence, distinguishes the C3 variant *S. thermophilum* from the thermally sensitive C3 variant found elsewhere. The relative proportion of this intragenomic variant exhibits variation between host coral genera in UAQ but, in some cases, also between colonies of the same genera (Fig. 5). Poritids showed substantial intra-generic variation with proportions of C3 Gulf sequences ranging from 1% to 17% of the individual’s C3 community while the acroporiids averaged a higher proportion of C3 Gulf sequences (~13%) but with less variability among samples (range: 12–15%). In agreement with the consistency among the ITS2 types, the proportion of this variant in the well-sampled genera did not change after the bleaching (Supplementary Table 3).

## Discussion

Corals in the UAQ lagoon are living at the thermal limits of present day corals and therefore provide an ideal opportunity to investigate the symbiont communities on extreme reefs. This study exploits the benefits of next generation amplicon sequencing to provide the first in-depth characterisation of southern PAG lagoonal symbiont communities. It is demonstrated that the symbiont community in a highly variable lagoon in the southern PAG is largely comprised of the C3 variant *S. thermophilum* and that the proportions of the dominant and background symbiont types remain stable despite severe bleaching induced by one of the most extreme temperature regimes reported for coral reefs.

### Extreme thermal environment

Corals growing in variable thermal environments have been shown to have greater tolerance to high temperatures^21^ than corals from more stable environments. While the summer maxima in these reefs can reach 35 °C^21^, the lagoonal reefs in UAQ are exposed to summer temperatures up to 37.5 °C as a result of the variability around high mean temperatures (>34 °C). The maxima experienced in UAQ are extreme even in the context of the southern PAG, where C3-dominated open water reefs only reached 35.2 °C during 2014 and records of temperatures of this magnitude are largely confined to anomaly events^53^. Considering that the UAQ reef temperatures dropped to 16.8 °C during the winter 2014–2015, the corals residing on these reefs must acclimate to one of the highest thermal ranges experienced by modern day corals.

### Symbiont communities dominated by *Symbiodinium thermophilum*

The capacity of corals to acclimatise to a thermal regime varying over 20 °C annually must require a thermally tolerant symbiont. Indeed, the ability for corals to survive in highly variable reefs in other regions has, in part, been attributed to symbioses with members of clade D^21^. Previous studies have shown on both local and regional scales that reefs with high thermal variability possess greater abundances of clade D symbionts than their counterparts in more stable thermal environments^20, 21^, as do corals on reefs with high thermal maxima^17, 26^. Clade D corals are also common to areas with high turbidity^54^. This would suggest that clade D ITS2 types should be relatively common in the UAQ lagoon, where temperature variability is high on annual (>20 °C range) and diurnal (July/August mean range ~3 °C) scales, and corals are exposed to extreme thermal stress in summer (max. 37.5 °C). In particular, the clade D species *S. trenchi* might be expected in the UAQ lagoon as it is a widespread, thermally tolerant opportunist that is found on other reefs in the region^12, 55^. However, our results show that clade D zooxanthellae are a relatively rare component of the symbiont community in corals in UAQ (<1% occurrence). Here, we show that the reefs in the lagoon are characterised by low symbiont diversity (only 3 functional OTUs present), with the coral community largely dominated by clade C3-type symbionts (97% occurrence), a pattern that was consistent across an extreme bleaching event.

The dominant OTU_C3 symbionts found in UAQ belong to the recent described species, *S. thermophilum*. This assertion is supported by the presence of the characteristic intragenomic ITS2 variant within all colonies, although variation in the proportion of this ITS2 sequence type among coral genera suggests there may be further variation within this new species. While the superior thermal tolerance of *S. thermophilum* has been supported by field surveys and laboratory experiments under relatively stable thermal regimes^19, 30^, the observations from this study suggest that this symbiont also has the capacity to withstand extreme thermally variable environments. In contrast, the rarity of clade D, and notable absence of *S. trenchi* in UAQ under conditions where it should thrive, implies that the lagoon environment here and the wider southern Gulf is largely unsuitable for clade D dominance, as D symbiont types (including *S. trenchi*) are only found in abundance in the thermally more benign northern PAG and Gulf of Oman^17, 27–29, 55^. While the extremes in temperature may cause the outperformance of *S. thermophilum* over clade D symbionts, the hypersaline environment may also apply a strong selective pressure to symbiont communities. This selective pressure potentially favours *S. thermophilum*, which appears adapted to higher salinity environments^29^.

Bleaching was widespread and recorded in all genera on UAQ lagoonal reefs in September 2014. The severe bleaching could have provided an opportunity for a shift in UAQ corals’ symbiont community to clade D, as these ITS2 types have been observed to opportunistically dominate corals after bleaching events due to their high thermal tolerance^4, 12, 13^. Adaptive bleaching could have occurred through switching or shuffling as clade D symbionts were present on the reef prior to bleaching and were present in background levels in individuals from all coral genera, respectively. Nevertheless, the bleaching experienced on UAQ lagoon reefs did not induce changes in the composition of the symbiont communities and no increase in the relative abundance of OTU_D1 was found. In fact, post-bleaching sampling in the genera where clade D was dominant prior to bleaching revealed that *Stylophora* colonies had suffered complete mortality (e.g. Supplementary Figure 1), however, they were a relatively minor component of the community prior to bleaching. It is apparent that despite being present on the reef prior to severe and widespread bleaching, this putatively opportunistic symbiont was unable to take advantage. Nevertheless, it is important to consider that the clade D symbionts present (e.g. D1-4-6) may not possess the thermal tolerance traits of other D types such as *S. trenchi* (D1-4)^7^. We postulate that the absence of adaptive bleaching results from the absence of a more tolerant symbiont than the *S. thermophilum* dominating these symbiont communities. In agreement with Stat and coworkers^18^, our observations from multiple coral genera with different symbiont transmission modes support the notion that changing the symbiont community in favour of D types may not be essential for resilience and recovery from extreme thermal events.

### Wider implications

The rate of temperature increase due to climate change could exceed the rate of adaptation by corals and therefore the capacity for modern day coral symbioses to acclimate is of paramount importance. The presence of reefs in an environment as extreme as the UAQ lagoon is a positive indicator for the potential of corals to cope with the increased temperatures forecast for reefs globally. The *S. thermophilum* dominated community observed here shows capacity for resilience to high thermal variability and extreme events, in addition to persistence under high mean summer temperatures. As symbionts can improve host thermal tolerance^4^, the possible export of these symbionts reported in the UAQ lagoonal reefs holds promise for the acquisition of southern PAG corals’ thermal tolerance by reefs elsewhere. Nevertheless, this will require further study as the fitness of *S. thermophilum* may be lost outside of the PAG’s unique conditions, particularly in normal oceanic salinities^29^. The coral-algal symbioses in UAQ appear to living close to their thermal limits as the extreme summer temperatures on these reefs caused widespread bleaching and did not select for an alternative symbiont type. Considering that regional sea surface temperatures have increased by 0.5 °C per decade since the 1980s^56^, further increases to temperature at this rate may soon push these corals beyond their threshold for survival in the near future. Consequently, the long - term persistence of this important source of thermally tolerant genotypes is at risk.

## Electronic supplementary material

Supplementary Material
