# Supplementary material for: Symbiont community stability through severe coral bleaching in a thermally extreme lagoon
Source: Sci Rep. 2017 May 25;7:2428. doi: 10.1038/s41598-017-01569-8 (PMC5445074; doi:10.1038/s41598-017-01569-8)
Supplement: Supplementary file 1 — Supplementary Material [file 41598_2017_1569_MOESM1_ESM.pdf]

Symbiont community stability through severe coral bleaching in a thermally extreme lagoon

Smith EG[1*], Vaughan GO[1], Ketchum RN[1], McParland D[1], Burt JA[1]

1. Centre of Genomics and Systems Biology, New York University Abu Dhabi, PO Box 129188, U.A.E.

*Corresponding author: edsmith@nyu.edu

Supplementary Material

	Average	Maximum	Minimum	Range
**UAQ1 vs Saadiyat**	*t = -0.902*<br>*df = 87*<br>*p = 0.37*	*t = 11.034*<br>*df = 89*<br>*p < 0.01*	*t = -8.408*<br>*df = 78*<br>*p < 0.01*	*t = 23.461*<br>*df = 63*<br>*p < 0.01*

Supplementary Table 1. Statistical parameters for pairwise t-tests comparing the thermal environment in the Umm al Quwain lagoon to the open water Saadiyat reef.

	UAQ1	UAQ2
**Platygyra**	*68*	*361*
**Porites**	*18*	*121*
**Cyphastrea**		*76*
**Dipsastraea**		*13*

Supplementary Table 2. Total CPCe counts for each coral genera, at each site, that were used for calculation of bleaching frequency.

	Proportion of C3 Gulf June vs December
**Acropora**	*Z=-1.173*<br>*p = 0.24*
**Cyphastrea**	*Z=0.174*<br>*p = 0.86*
**Porites**	*Z=-0.285*<br>*p = 0.78*
**Platygyra**	*Z=-1.072*<br>*p = 0.28*

Supplementary Table 3. Statistical parameters for two sample Mann Whitney U tests comparing the relative proportion of C3-Gulf sequences within taxa between time points.

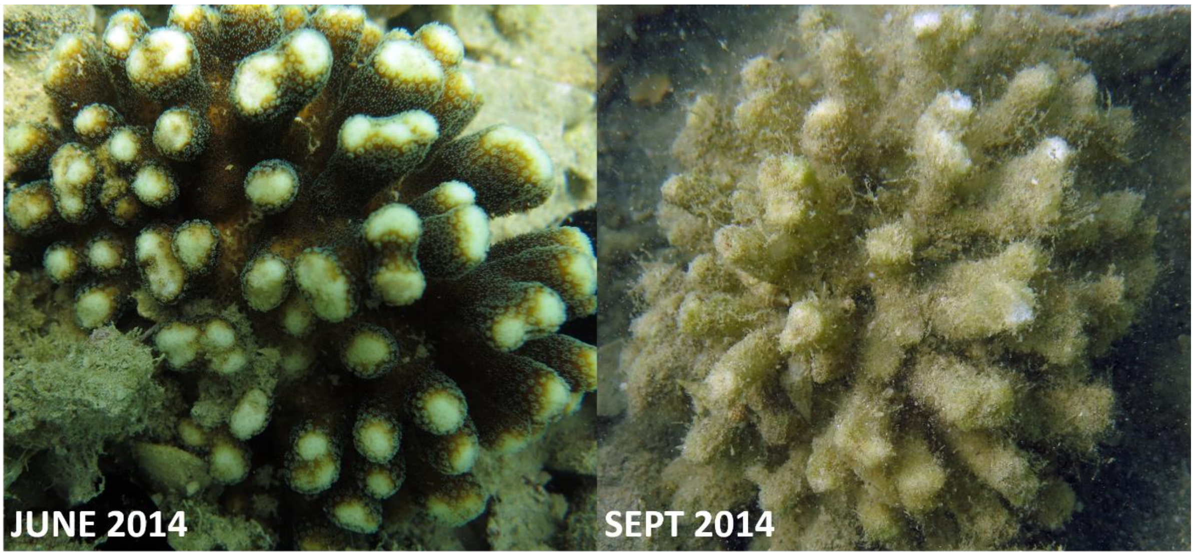

Supplementary Figure 1. Example of a *Stylophora* colony that had suffered complete mortality during summer 2014.

```
OTU_A1          GTGAACCAATGGCCTCTTGAACGTGCATTGCGCTCTTGGGATATGCCTGAGAGCATGTCT
OTU_A_PSEUDO1   GTGGCCCAATGGCCTCTTGAACGTGCATTGCGCTTTTGGGATATGCCAGAGAGCATGTCT
OTU_A_PSEUDO2   GTGAACCAATGGCCTCTTGAACGTGCATTGCGCTCTTGGGATATGCCTGAGAGCATGTCT


OTU_A1          GCTTCAGTGCTTCTACTTTCATTTT---CTGCTGCTCTTGTTATCAGGAGCAGTGTTGCT
OTU_A_PSEUDO1   GCTGCAGTTTTTCTACTTTCATTTT---CTGCTGCTCTTGTTATCAGAAGCAATGTTGCT
OTU_A_PSEUDO2   GCTTCAGTGCTTCTAGTCTCAATTTGTCCTGCTGCTCCTTCCAGTAGGGGTAGTGCTGCT


OTU_A1          GCATGCTTCTGC-AAGTGGCACTGGCATGCTAAATATCAAGTTTTGCTTGCTGTTGTGAC
OTU_A_PSEUDO1   GCATGCTTCTGC-AAGTGGCACTGGCATGCTAAA--------------------------
OTU_A_PSEUDO2   GCATGCTACTACTTCAAAGCACTGGCATGCTGAGTATTAAGTTTCGCCCACTGGTTTGAC


OTU_A1          TGATCAACATCTCATGTCGTTTCAGTTGGCGAAACAAAAGCTCATGTGTGTTCTTAACAC
OTU_A_PSEUDO1   ----------------TCGTTTGAGTTGGCGAAACAAAAGCTCAGGTGTGTTCTTAACAC
OTU_A_PSEUDO2   TGATCAACATCAAATGTCTTGTCGGCTGGGCGAATGA---CCCAAAAGTGTTTTGAACAC


OTU_A1          TTCCTA
OTU_A_PSEUDO1   TTCCTA
OTU_A_PSEUDO2   TTCCTA
```

Supplementary Data 1. Clade A OTU alignment. Alignment of clade A OTUs at 97% similarity, including putative pseudogenes.

```
OTU_C3          CTCTTGGGATTTCCTGAGAGTATGTCTGCTTCAGTGCTTAACTTGCCCCAACTTTGCAAG
OTU_C_PSEUDO    CTCTTGGGATTTCCTGAGAGTATGTCTGCTTCAGTGCTTAACTTGCCCCAACTTTGCAAG


OTU_C3          CAGGATGTGTTTCTGCCTTGCGTTCTTATGAGCTATTGCCCTCTGAGCCAATGGCTTGTT
OTU_C_PSEUDO    CAGGATGTGTTTCTGCCTTGCGTTCTTATGAGCTATTGCCCTCTGAGCCAATGGCTTGTT


OTU_C3          AATTGCTTGGTTCTTGCAAAATGCTTTGCGCGCTGTTATTCAAGTTTCTACCTTCGTGGT
OTU_C_PSEUDO    AATTGCTTGGTTCTTGCAAAA---------------------------------------


OTU_C3          TTTACTTGAGTGACGCTGC--------TCATGCTTGCAACCGCTGGGATGCAGGTGCATG
OTU_C_PSEUDO    ---------GTGACGCTGCGACGCTGCTCATGCTTGCAACCGCTGGGATGCAGGTGCATG


OTU_C3          CCTCTA
OTU_C_PSEUDO    CCTCTA
```

Supplementary Data 2. Clade C OTU alignment. Alignment of clade C OTUs at 97% similarity, including putative pseudogene.

```
OTU100_D1       TGGGACTTCCTGAGAGTATGTTTGCTTCAGTGCTTATTTTACCTCCTTGCAAGGTTCTGT
OTU100_D18      TGGGACTTCCTGAGAGTATGTTTGCTTCAGTGCTTATTTTACCTCCTTGCAAGGTTCTGT
OTU100_D6       TGGGACTTCCTGAGAGTATGTTTGCTTCAGTGCTTATTTTACCTCCTTGCAAGGTTCTGT
OTU100_D4       TGGGACTTCCTGAGAGTATGTTTGCTTCAGTGCTTATTTTACCTCCTTGCAAGGTTCTGT

OTU100_D1       CGCAACCTTGTGCCCTGGCCAGCCATGGGTTAACTTGCCCATGGCTTGCTGAGTAGTGAT
OTU100_D18      CGCAACCTTGTGCCCTGGCCAGCCATGGGTTAACTTGCCCATGGCTTGCTGAGTAGTGAT
OTU100_D6       CGCAACCTTGTGCCCTGGCCAGCCACGCGTTAACTTGCCCATGGCTTGCTGAGTAGTGAT
OTU100_D4       CGCAACCTTGTGCCCTGGCCAGCCACGGGTTAACTTGCCCATGGCTTGCTGAGTAGTGAT

OTU100_D1       CTTTTAGAGCAAGCTCTGGCACGCTGTTGTTTGAGGCAGCCTATATTGAGGCTATTTCAA
OTU100_D18      CTTTTAGAGCAAGCTCTGGCACGCTGTTGTTTGAGGCAGCCTATATTGAGGCTATTTCAA
OTU100_D6       CTTTTAGAGCAAGCTCTGGCACGCTGTTGTTTGAGGCAGCCTATATTGAGGCTATTTCAA
OTU100_D4       CTTTTAGAGCAAGCTCTGGCACGCTGTTGTTTGAGGCAGCCTATATTGAGGCTATTTCAA

OTU100_D1       ATGACGTTGCTACAAGCTTGATGTGTCCTTCTGCGCCGTTGCGCATCCCATA
OTU100_D18      ATGACGTTGCTACAAGCTTGATGTGTCCTTCTGCGCCGTCGCGCATTCCATA
OTU100_D6       ATGACGTTGCTACAAGCTTGATGTGTCCTTCTGCGCCGTTGCGCATCCCATA
OTU100_D4       ATGACGTTGCTACAAGCTTGATGTGTCCTTCTGCGCCGTTGCGCATCCCATA
```

Supplementary Data 3. Clade D alignment. Alignment of clade D OTUs at 100% similarity level that are present in greater than 10% abundance within an individual.
